# Evolutionary transformation of mouthparts from particle-feeding to piercing carnivory in Viper copepods: Review and 3D analyses of a key innovation using advanced imaging techniques

**DOI:** 10.1186/s12983-019-0308-y

**Published:** 2019-08-22

**Authors:** Tomonari Kaji, Chihong Song, Kazuyoshi Murata, Shigenori Nonaka, Kota Ogawa, Yusuke Kondo, Susumu Ohtsuka, A. Richard Palmer

**Affiliations:** 1 Department of Biological Sciences, University of Alberta, Edmonton, AB T6G 2E9 Canada; 2 Allgemeine & Spezielle Zoologie, Institut fur Biowissenschaften, Universität Rostock, 18055 Rostock, Germany; 3 National Institute for Physiological Sciences, Okazaki, Aichi 444-8585 Japan; 4 National Institute for Basic Biology, Okazaki, Aichi 444-8585 Japan; 5 Setouchi Field Science Center, Graduate School of Integrated Sciences for Life, Hiroshima University, 5-8-1 Minato-machi, Takehara, Hiroshima 725-0024 Japan; 6 Biosystematics Laboratory, Faculty of Social and Cultural Studies, Kyushu University, Okazaki, Hiroshima Japan

**Keywords:** Feeding strategies, Functional morphology, Morphological novelty, Appendage innovation, Crustacea, Calanoida, Venom injection, Adaptive radiation, Evolutionary history, Phylogenetic relations, Secretory glands

## Abstract

**Background:**

Novel feeding adaptations often facilitate adaptive radiation and diversification. But the evolutionary origins of such feeding adaptations can be puzzling if they require concordant change in multiple component parts. Pelagic, heterorhabdid copepods (Calanoida) exhibit diverse feeding behaviors that range from simple particle feeding to a highly specialized form of carnivory involving piercing mouthparts that likely inject venom. We review the evolutionary history of heterorhabdid copepods and add new high-resolution, 3D anatomical analyses of the muscular system, glands and gland openings associated with this remarkable evolutionary transformation.

**Results:**

We examined four heterorhabdid copepods with different feeding modes: one primitive particle-feeder (*Disseta palumbii*), one derived and specialized carnivore (*Heterorhabdus subspinifrons*), and two intermediate taxa (*Mesorhabdus gracilis* and *Heterostylites longicornis*). We used two advanced, high-resolution microscopic techniques — serial block-face scanning electron microscopy and two-photon excitation microscopy — to visualize mouthpart form and internal anatomy at unprecedented nanometer resolution. Interactive 3D graphical visualizations allowed putative homologues of muscles and gland cells to be identified with confidence and traced across the evolutionary transformation from particle feeding to piercing carnivory. Notable changes included: a) addition of new gland cells, b) enlargement of some (venom producing?) glands, c) repositioning of gland openings associated with hollow piercing fangs on the mandibles, d) repurposing of some mandibular-muscle function to include gland-squeezing, and e) addition of new muscles that may aid venom injection exclusively in the most specialized piercing species. In addition, live video recording of all four species revealed mandibular blade movements coupled to cyclic contraction of some muscles connected to the esophagus. These behavioral and 3D morphological observations revealed a novel injection system in *H. subspinifrons* associated with piercing (envenomating?) carnivory.

**Conclusions:**

Collectively, these results suggest that subtle changes in mandibular tooth form, and muscle and gland form and location, facilitated the evolution of a novel, piercing mode of feeding that accelerated diversification of the genus *Heterorhabdus*. They also highlight the value of interactive 3D animations for understanding evolutionary transformations of complex, multicomponent morphological systems.

**Electronic supplementary material:**

The online version of this article (10.1186/s12983-019-0308-y) contains supplementary material, which is available to authorized users.

## Background

Key evolutionary innovations are adaptations that facilitate rapid and sometimes extensive diversification of lineages within which they arise [1, 2]. Familiar examples include insect wings [3], bird feathers [4], biting jaws of vertebrates [5], and pharyngeal jaws in cichlid fish [6]. However, to be fully functional, many such key innovations require concordant changes in multiple body components, which can yield controversies about the order and integration of the evolutionary transformations that ultimately gave rise to them (e.g., [5]).

**Fig. 1:**
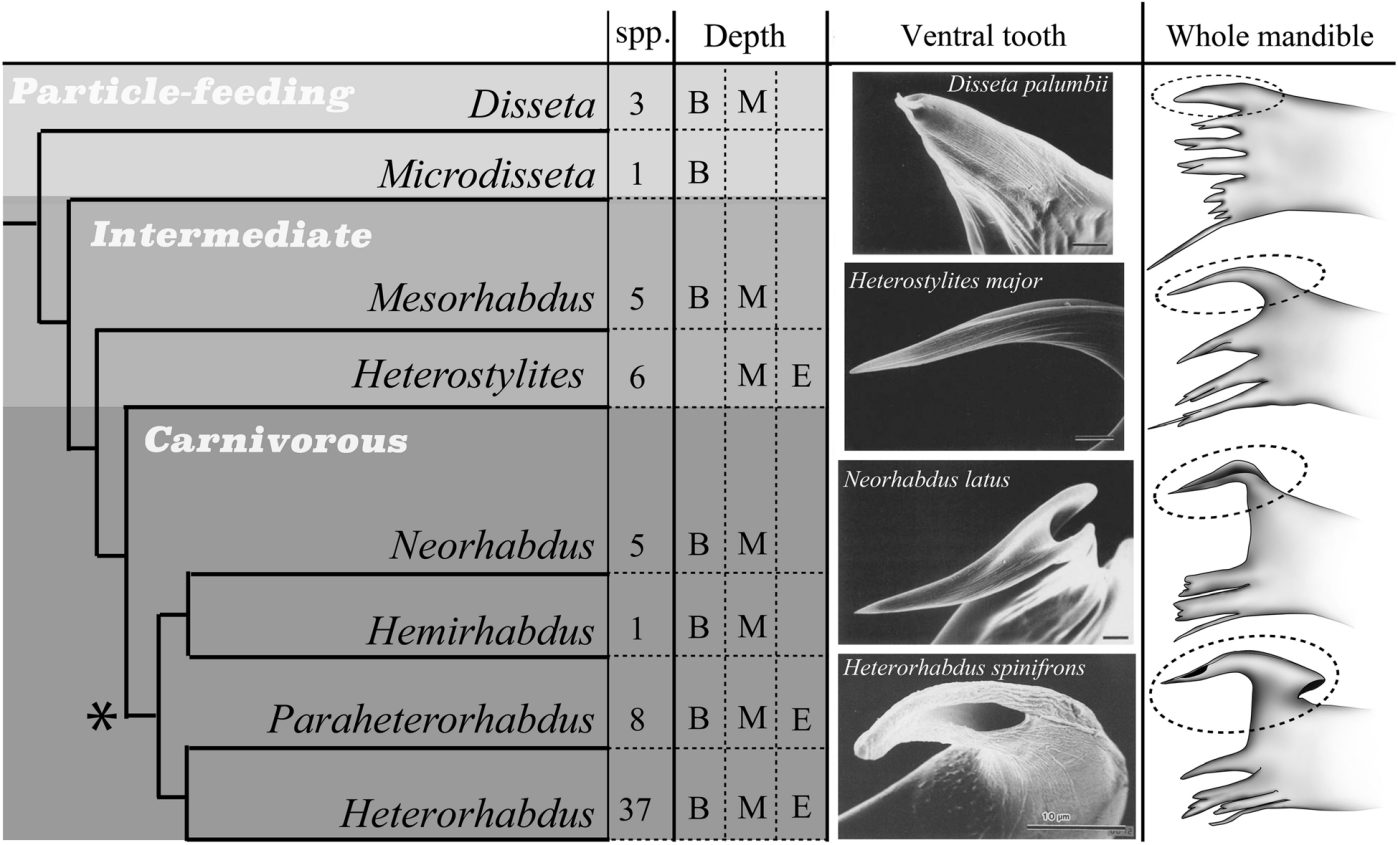
Overview of evolutionary relations, feeding modes, species diversity, depth distributions, and mandible and ventral tooth form of Viper copepods (Heterorhabdidae, Calanoida). Phylogeny after Ohtsuka et al. [9] and (Hirabayashi et al. [10]), feeding modes from Ohtsuka et al. [9], species diversity from WoRMS (http://www.marinespecies.org/, July 2017), ocean depth ranges from Ohtsuka et al. ([9]; B- Bathypelagic, M- Mesopelagic, E- Epipelagic), SEM images from Ohtsuka et al. [9], drawings by TK. Asterisk: inferred origin of poison-injection system (Hirabayashi et al. [10]), Dashed circle: ventral tooth

A less familiar, but no less fascinating, key innovation evolved in pelagic ‘Viper’ copepods (Heterorhabidae, Calanoida): mandibles bearing tubular, hypodermic-needle-like structures (e.g., *Heterorhabdus*, Fig. 1) that are thought to inject venom secreted from openings of enlarged glands located in the upper lip (labrum) [7]. This fang-bearing mandible differs considerably in form from the mouthparts of typical particle-feeding copepods such as Calanidae and Paracalanidae [8]. It also differs from primitive particle feeding heterorhabid copepods [9, 10], all of which possess mandibles with macerating or cutting teeth (e.g., *Disseta,* Fig. 1). These unique hollow fangs of *Heterorhabdus* were the first potentially envenomating structure to be reported from crustaceans [9, 11, 12].

Piercing carnivory in Viper copepods (asterisk, Fig. 1) qualifies as a key innovation because it is associated with both a) accelerated diversification, and b) expansion of ecological (depth) range. Of eight heterorhabdid genera [13], the two with the most derived forms of piercing carnivory (*Heterorhabdus* and *Paraheterorhabdus*) include nearly 2/3 of all heterorhabdid species [9, 10] (Fig. 1; WoRMS 2018). In addition, these two genera span the widest depth range of all heterorhabdid genera, which mostly occur in the deep sea [9] [mesopelagic (M) or bathypelagic (B), Fig. 1].

Previous morphological and phylogenetic studies showed that feeding habits changed from particle feeding to carnivory in the Heterorhabdidae [7, 9, 10] (Fig. 1). The basally branching genera *Disseta* and *Microdisseta* are essentially particle-feeders, while the derived genera *Neorhabdus*, *Hemirhabdus*, *Paraheterorhabdus* and *Heterorhabdus* are carnivores. *Mesorhabdus* and *Heterostylites* are intermediate between these extremes.

Mandible form changed dramatically associated with these diet changes [9]. The ventral-most mandibular tooth of typical particle-feeders (e.g., *Disseta* and *Microdisseta*) is unspecialized and similar to that of other particle feeding calanoid copepods (Fig. 1). In intermediate taxa, the ventral tooth is enlarged (e.g., *Mesorhabdus* and *Heterostylites*; Fig. 1). In one intermediate taxon the elongate ventral tooth possesses a fine groove (*Heterostylites*; Fig. 1). The ventral tooth in carnivorous taxa bears a massive groove or is partly tubular in some taxa (*Hemirhabdus* and *Neorhabdus*; Fig. 1). In the most diverse and ecologically widely distributed Viper copepods (*Paraheterorhabdus* and *Heterorhabdus*) the ventral mandibular tooth forms a completely enclosed tube (Fig. 1).

The effectiveness of piercing carnivory depends not only on mandible form, but also on a) glands that secrete substances to facilitate prey capture and ingestion, and b) muscles that move the mandibular gnathobase. Three sets of gland openings in the upper lip (labrum) are associated with secretory cells in all heterorhabdid copepods [7, 9]. Each set is thought to be associated with a different set of glands. However, details of gland structure are known only for the carnivore *Heterorhabdus* [7], and nothing is known about the muscles that control mandibular motion in any heterorhabdid copepod.

To better understand the fine structure and spatial relations among glands and muscles within the mouthparts of Viper copepods, we utilized two advanced, high-resolution imaging methods — serial block face scanning electron microscopy (SBF-SEM) and two-photon excitation microscopy — to produce 3D nanometer-scale reconstructions of the external and internal morphology of the labrum (=upper lip) and paragnath (=lower lip) of heterorhabdid species from four genera: *Disseta palumbii* Giesbrecht, 1889*, Mesorhabdus gracilis* Sars, 1907*, Heterostylites longicornis* (Giesbrecht, 1889)*,* and *Heterorhabdus subspinifrons* Tanaka, 1964. We also video-recorded mouthpart movement in live specimens of all four taxa to clarify muscle function. Finally, to reconstruct the evolutionary history of this innovative feeding mode, we compared putative homologues of component elements (muscles, glands, gland openings) among all four genera and discuss character variation across the phylogenetic tree of heterorhabdid copepods.

## Results

**Fig. 2:**
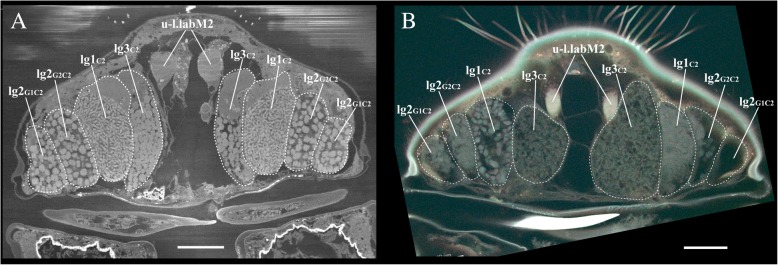
Comparison of two different scan methods to reconstruct a transverse plane of the anterior part of the labrum from two separate individuals of *Mesorhabdus gracilis*. Dashed lines identify the boundaries of the labeled glands (see abbreviations list and Table 1 for gland and muscle names and abbreviations). **a** Scan from SBF-SEM. **b** Scan from two-photon excitation microscopy. Note the significant differences in appearance of gland contents between these two individuals, which suggests that high-resolution images of gland contents may not be very informative phylogenetically

Both state-of-the-art SBF-SEM microscopy [14] and two-photon excitation microscopy [15], combined with associated image-analysis technologies, yielded full 3D perspectives — at nano-scale resolution — of the glands and muscles in the mouthparts of the heterorhabdid species studied. Although these two methods are based on different principles, and use different types of fixative, the results were similar for both (Fig. 2). Sections from two different individuals of *Mesorhabdus gracilis* (Fig. 2) show planes of four pairs of glands (dashed outlines), and planes of one pair of muscles, which correspond nicely between the two pictures. Both imaging methods clearly show the same spatial relationships of glands and muscles. Gland contents, however, appeared to differ somewhat between methods. For example, gland lg1C2 in the SBF-SEM scan (Fig. 2a) appeared to be filled with tiny and flattened disc-shaped granules, whereas in the two-photon excitation microscopy scan (Fig. 2b) the granules appeared to be rather big and more rounded in shape. Curiously, gland contents also appeared to differ between sides even within a single specimen (compare contents of gland lg1C2 on the left and right side of Fig. 2b).

Numerous muscles and glands are associated with the mouthparts examined. All of the descriptive terms used here to refer to morphological units of muscles and glands do not imply any homology hypothesis (see [29] for a discussion of homology-free terminology in morphological description). Homology hypotheses for these descriptive terms are outlined explicitly in Table 1 and discussed in detail in the Discussion.

**Table 1:** Homology hypotheses for muscles and glands and their formal descriptions, following the scheme adopted by [30]. Each morphological unit in the left-most column is inferred to be homologous among all four species but has spatial relations, connections and constituents as indicated under each species

Hypothetical homologues	Category of descriptions	*Disseta palumbii*	*Mesorhabdus gracilis*	*Heterostylites longicornis*	*Heterorhabdus subspinifrons*
MUSCLES					
Esophageal Sphincters (esoS)	Spatial relationships	surround esophagus opening	surround esophagus opening	surround esophagus opening	surround esophagus opening
	Connections	esophagus	esophagus	esophagus	esophagus
Forehead-Esophageal Dilator Muscles (for-eso.dM)	Spatial relationships	anterior to esophagus, dorsal to lab-eso.dM2	anterior to esophagus, dorsal to lab-eso.dM2 and 4	anterior to esophagus, dorsal to lab-eso.dM2	anterior to esophagus, dorsal to lab-eso.dM2
	Connections	esophagus, forehead	esophagus, forehead	esophagus, forehead	esophagus, forehead
	Constituents	4 pair of muscles	several muscle bundles	several muscle bundles	several muscle bundles
Labrum-Esophageal Dilator Muscles 1 (lab-eso.dM1)	Spatial relationships	anterior to esophagus, lateral to for-eso.dM	anterior to esophagus, dorsal to lg1, lateral to for-eso.dM	anterior to esophagus, lateral to for-eso.dM	anterior to esophagus, lateral to lg3, lateral to for-eso.dM2
	Connections	esophagus, anterior	esophagus, anterior	esophagus, anterior	esophagus, anterior
	Constituents	−	−	−	−
Labrum-Esophageal Dilator Muscles 2 (lab-eso.dM2)	Spatial relationships	anterior to esophagus, dorsal to u-l.labM4, ventral to for-eso.dM	anterior to esophagus, dorsal to lab-eso.dM4, dorsal to lg1, ventral to for-eso.dM	anterior to esophagus, dorsal to lab-eso.dM4, ventral to for-eso.dM	anterior to esophagus, dorsal to lab-eso.dM4, medial to lg3, ventral to for-eso.dM
	Connections	esophagus, anterior labrum, u-l.labM1	esophagus, anterior labrum	esophagus, anterior labrum, u-l.labM1	esophagus, anterior labrum, u-l.labM1
Labrum-Esophageal Dilator Muscles 3 (lab-eso.dM3)	Spatial relationships	−	antero-ventral to esophagus, lateral to u-l.labM2, between lg1 and lg3, ventral to lab-eso.dM2	antero-ventral to esophagus, lateral to u-l.labM2, between lg1, lg2 and lg3, ventral to lab-eso.dM2	antero-ventral to esophagus lateral to u-l.labM2, between lg1 and lg3, ventral to lab-eso.dM2
	Connections	−	esophagus, lateral labrum	esophagus, lateral labrum	esophagus, lateral labrum
Labrum-Esophageal Dilator Muscles 4 (lab-eso.dM4)	Spatial relationships	antero-ventral to esophagus, ventral to lab-eso.dM2, dorsal to u-l.labM2	antero-ventral to esophagus, ventral to lab-eso.dM2, dorsal to u-l.labM2	antero-ventral to esophagus, ventral to lab-eso.dM2, dorsal to u-l.labM2	antero-ventral to esophagus, medial to lg3, ventral to lab-eso.dM2, dorsal to u-l.labM2
	Connections	esophagus, anterior labrum, u-l.labM2	esophagus, anterior labrum, u-l.labM 2	esophagus, anterior labrum, u-l.labM2	esophagus, anterior labrum, u-l.labM2
Lateral-Esophageal Dilator Muscles 1 (lat-eso.dM1)	Spatial relationships	antero-lateral to esophagus, lateral to for-eso.dM, dorsal to lab-eso.dM1	antero-lateral to esophagus, lateral to for-eso.dM, dorsal to lab-eso.dM1	antero-lateral to esophagus, lateral to for-eso.dM, dorsal to lab-eso.dM1	antero-lateral to esophagus, lateral to for-eso.dM, dorsal to lab-eso.dM1
	Connections	esophagus, antero-lateral body wall	esophagus, antero-lateral body wall	esophagus, antero-lateral body wall	esophagus, antero-lateral body wall
Lateral-Esophageal Dilator Muscles 2 (lat-eso.dM2)	Spatial relationships	lateral to esophagus	lateral to esophagus, dorsal to lg2	lateral to esophagus	ateral to esophagus, dorsal to lg1
	Connections	esophagus, antero-lateral body wall	esophagus, antero-lateral body wall	esophagus, antero-lateral body wall	esophagus, antero-lateral body wall
Paragnath Muscles (parM)	Spatial relationships	within paragnath	within paragnath	within paragnath	within paragnath, antero-dorsal to lg1C1
	Connections	anterior paragnath	anterior paragnath	anterior paragnath	anterior paragnath
	Constituents	2 pair of muscles	2 pair of muscles	2 pair of muscles	2 pair of muscles
Saggital Labral Muscles (s.labM)	Spatial relationships	−	−	−	anterior to posterior wall of labrum, medial to lg1C2, dorsal to lg3C2
	Connections	−	−	−	dorso-medial of the posterior wall of labrum, ventro-lateral of the posterior wall of labrum
Transversus Labral Muscle (t.labM)	Spatial relationships	dorsal to lg3 and lg1, ventral to lab-eso.dM3, between bundles of u-l.labM2	dorsal to lg3 and lg1, ventral to lab-eso.dM3, between bundles of u-l.labM2	dorsal to lg3 and lg1, ventral to lab-eso.dM3, between bundles of u-l.labM2	dorsal to lg3 and lg1, ventral to lab-eso.dM3, between bundles of u-l.labM2
	Connections	cuticular ridge from dorsal labrum	cuticular ridge from dorsal labrum	cuticular ridge from dorsal labrum	cuticular ridge from dorsal labrum
Upper-Lower Labral Muscles 1 (u-l.labM1)	Spatial relationships	medial to u-l.labM2, medial to lab-eso.dM4	medial to u-l.labM2, medial to lab-eso.dM4, lateral to legC1	medial to u-l.labM2, medial to lab-eso.dM4	medial to u-l.labM2, medial to lab-eso.dM4, medial to lg3C2
	Connections	anterior-medial labrum, ventro-medial of dorsal labrum	anterior-medial labrum, ventro-medial of dorsal labrum	anterior-medial labrum, ventro-medial of dorsal labrum	anterior-medial labrum, ventro-medial of dorsal labrum
Upper-Lower Labral Muscles 2 (u-l.labM2)	Spatial relationships	lateral to u-l.labM1, medial to lg3	lateral to u-l.labM1, medial to lg3	lateral to u-l.labM1, medial to lg3	lateral to u-l.labM1, medial to lg3
	Connections	anterior labrum, posterior labrum	anterior labrum, posterior labrum	anterior labrum, posterior labrum	anterior labrum, posterior labrum
GLANDS					
Labral Gland Type 1 (lg1)	Spatial relationships	medial to lb2, lateral to lb3, within labrum	medial to lb2, lateral to lb3, lateral to lab-eso.dM3, within labrum	dorsal to lb, lateral to lb3, lateral to lab-eso.dM3, within labrum	medial to lb2, lateral to lb3, lateral to lab-eso.dM1 and 3, lateral to s.labM, within labrum and paragnath
	Connections	postero-lateral of labrum	postero-lateral of labrum	postero-lateral of labrum	postero-lateral of labrum
	Constituents	lg1C1-4	lg1C1 and 2	lg1C1 and 2	lg1C1 and 2
Labral Gland Type 2 (lg2)	Spatial relationships	ventro-lateral to lg1	lateral to lg1	ventro-lateral to lg1, ventral to lg3, surround lab-eso.dM3	lateral to lg1, lateral to s.labM
	Connections	postero-ventral edge of labrum	postero-ventral edge of labrum	postero-ventral edge of labrum	postero-ventral edge of labrum
	Constituents	lg2G1C1-4, lg2G2C1 and 2, lg2G3C1-3	lg2G1C1 and 2, lg2G2C1 and 2	lg2G1C1 and 2, lg2G2C1 and 2	lg2C1-3
Labral Gland Type 3 (lg3)	Spatial relationships	medial to lb1, lateral to u-l.labM2	medial to lb1, lateral to u-l.labM2	medial to lb1, lateral to u-l.labM2, medial to lab-eso.dM3	lateral to u-l.labM2, lateral to lab-eso.dM2 and 4, ventro-lateral to for-eso.dM, medial to lab-eso.dM1 and 3
	Connections	posterior labrum	posterior labrum	posterior labrum	posterior labrum
	Constituents	lg3C1 and 2	lg3C1 and 2	lg3C1 and 2	lg3C1-3
Labral Epidermal Glands (leg)	Spatial relationships	−	ventral to u-l.labM1	ventral to u-l.labM1	ventral to u-l.labM1, ventral to lg3
	Connections	−	antero-ventral labrum	antero-ventral labrum	antero-ventral labrum
	Constituents	−	leg1 and 2	leg1-8	leg1-3
Paragnathal Epidermal Glands (peg)	Spatial relationships	−	−	lateral to parM, beside lateral wall of paragnath	lateral to lg1C1, beside lateral wall of paragnath
	Connections	−	−	ventro-lateral paragnath	postero-lateral paragnath
	Constituents	−	−	peg right 1-3, left 1-3	pegG1C1 and 2, pegG2C1 and 2

### Gland morphology and arrangement

**Fig. 3:**
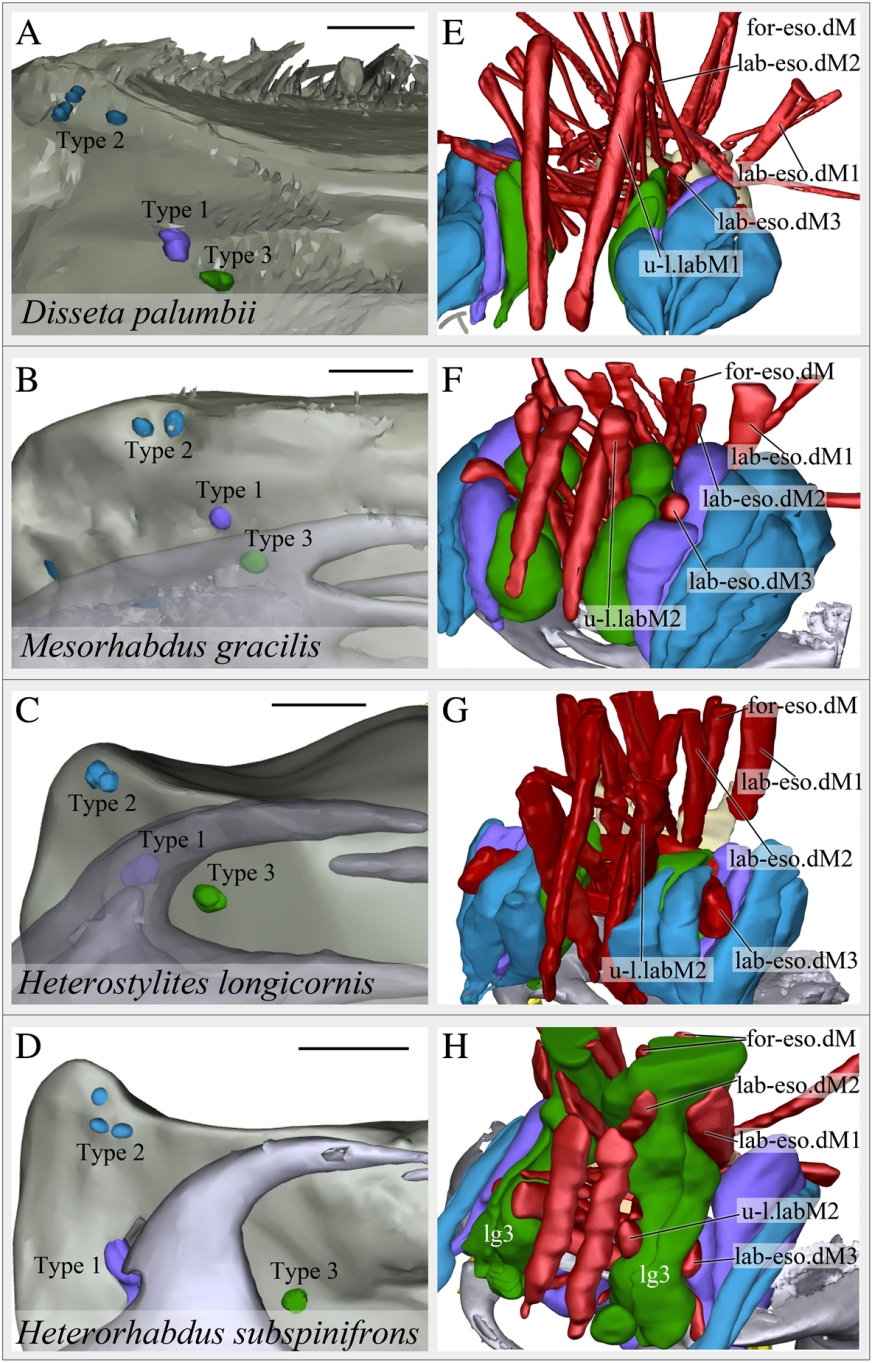
Mandible form, gland openings and anatomical microstructure of the muscle and gland systems associated with the mouthparts of heterorhabdid copepods. Left panels show the distribution of gland openings on the labrum (as viewed from the posterior, dorsal side down). Right panels show the detailed configuration of muscles and glands in the labrum (from an antero-ventro-lateral viewing perspective; see Fig. 3 for complete, interactive 3D viewing options of the internal anatomy). **a**, **e** *Disseta palumbii*. **b**, **f** *Mesorhabdus gracilis*. **c**, **g** *Heterostylites longicornis*. **d**, **h** *Heterorhabdus subspinifrons*. See abbreviations list and Table 1 for gland and muscle names and abbreviations. Color codes: purple- Labral Gland Type 1, blue- Labral Gland Type 2, green- Labral Gland Type 3, red- muscles, grey- mandibles. Scale bars, 50 μm for (**a**), 25 μm for (**b**-**d**)

We adopted labral gland terms from Nishida and Ohtsuka [7], where gland cells were divided into three “Types” according to the arrangement of gland openings (Fig. 3a-d). We use the same terminology here, but apply these terms differently except for *Heterorhabdus subspinifrons.* The arrangement of gland openings is essentially the same as reported previously [7], but we found an extra opening of labral gland Type 2 in *Disseta palumbii*: two openings were reported earlier [7], but we found a third (Fig. 3a).

**Fig. 4:**
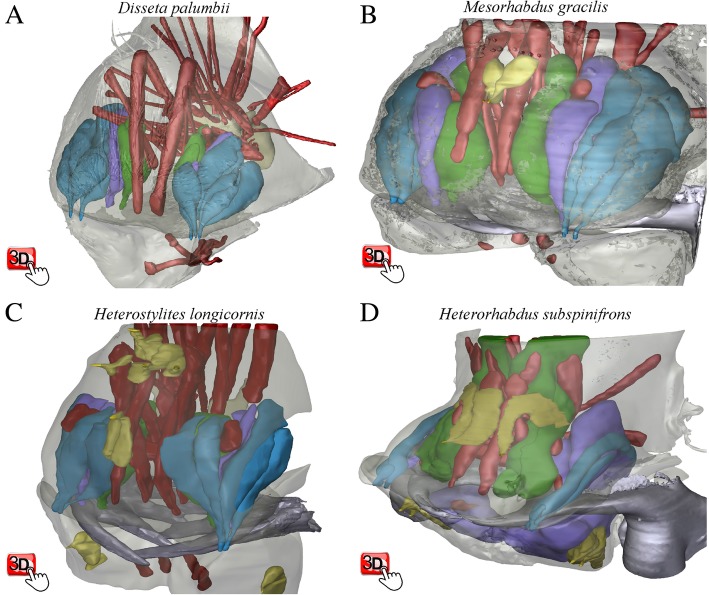
Three-dimensional surface models of whole muscles and glands in the labrum and paragnath of all four heterorhabdid species: **a**) *Disseta palumbii,* **b**) *Mesorhabdus gracilis*, **c**) *Heterostylites longicornis*, D) *Heterorhabdus subspinifrons*. The PDF version of the paper contains interactive 3D content that can be activated by clicking on each figure panel in Adobe Reader. To view/exclude individual drawing elements: 1) click on a figure panel to activate it, 2) click on the “Toggle Model Tree” icon in the 3D tool bar to display viewing options, and 3) check/uncheck drawing elements to include/exclude specific elements. In any view, use the scroll function to zoom in/out and click/drag the cursor to rotate the view. To observe the specific views referred to in the text, select the named view from list of views in the “Model Tree” side bar (for a detailed explanation of interactive 3D viewing functions, see Additional file 1: Figure S1). Color codes as in Fig. 2, except for yellow- labral gland and paragnathal epidermal gland, and tan- esophagous. Note: the orientation of the X-Y-Z axis indicators are arbitrary for each panel and are not comparable among panels

Even though gland openings were readily identified and easy to homologize among taxa, the size, shape and configuration of gland cells differed considerably among the four genera. In the particle feeding *D. palumbii*, gland cells are located postero-ventrally in the labrum, and are not associated with muscles (Fig. 3a, e: see Additional file 1: Figure S1 for viewing instructions for the interactive 3D-pdf images). In *Mesorhabdus gracilis* (intermediate feeding mode), the labrum is almost fully packed with labral gland cells and parts of these cells intercalate between the muscles lab-eso.dM3 and u-l.labM2 (Fig. 3b and f). In *Heterostylites longicornis* (intermediate feeding mode), labral gland cells are located at the posterior half of the labrum, and half of the cells are stacked between muscles lab-eso.dM3 and u-l.labM2 (Fig. 3g and c). Significantly, in the piercing carnivore, *H. subspinifrons*, all of the labral gland cells are highly extended anteriorly: a) Type 3 gland cells are enveloped by three muscles u-l.labM2, lab-eso.dM1–4 and for-eso.dM (Fig. 3h and d: click on the view “Labral Gland Type 3 and muscles” in the interactive 3D-PDF, Fig. 4d), b) Type 2 gland cells extend up to the posterior margin of the paragnath (Fig. 4d), and c) Type 1 gland cells are inflated, and posteriorly elongated into the paragnath (Fig. 4d). The total number of cells in gland Types 1–3 also differed among these genera (Table 1). *Disseta palumbii* has 15 pairs of cells, but *M. gracilis*, *H. longicornis*, and *H. subspinifrons* have only 8 pairs (Table 1). Type 1 and Type 3 glands were largest in the piercing carnivore, *H. subspinifrons* (Fig. 4d), but all three types were well-developed in the intermediate feeding-mode *M. gracilis* (Fig. 4b).

Our observations of cell numbers and orientation in each gland differ somewhat from Nishida and Ohtsuka [7]. They reported “*Type 1 and 3 labral glands have two secretory cells...Type 2 labral glands and the paragnathal gland have one secretory cell*” in *Heterorhabdus abyssalis, H. pacificus, H. papilliger,* and *H. spinifrons*. However, our observation of *H. subspinifrons* confirmed two cells in Type 1 glands, but revealed three cells in each of Types 2 and 3 (Fig. 4d, Table 1). Regarding cell structures, Type 2 gland cells were previously considered to be anteriorly elongate cells along the labral wall, and Type 3 gland cells as small cells located within the posterior side of the labrum [7]. However, our observations revealed that Type 2 gland cells extend toward the paragnath, and that the dramatically inflated Type 3 gland cells were directed anteriorly, reaching all the way to the forehead.

The arrangement of gland openings also differed between the carnivore *Heterorhabdus* and the non-carnivore taxa. The openings line up nearly in a straight line in *D. palumbii, M. gracilis* and *H. longicornis*, but the opening for Type 1 lies far off the line in *H. subspinifrons* (Fig. 3a-d). Significantly, the opening for the Type 1 gland in *H. subspinifrons* lies directly at the posterior end of the hollow fang (Fig. 4d).

**Fig. 5:**
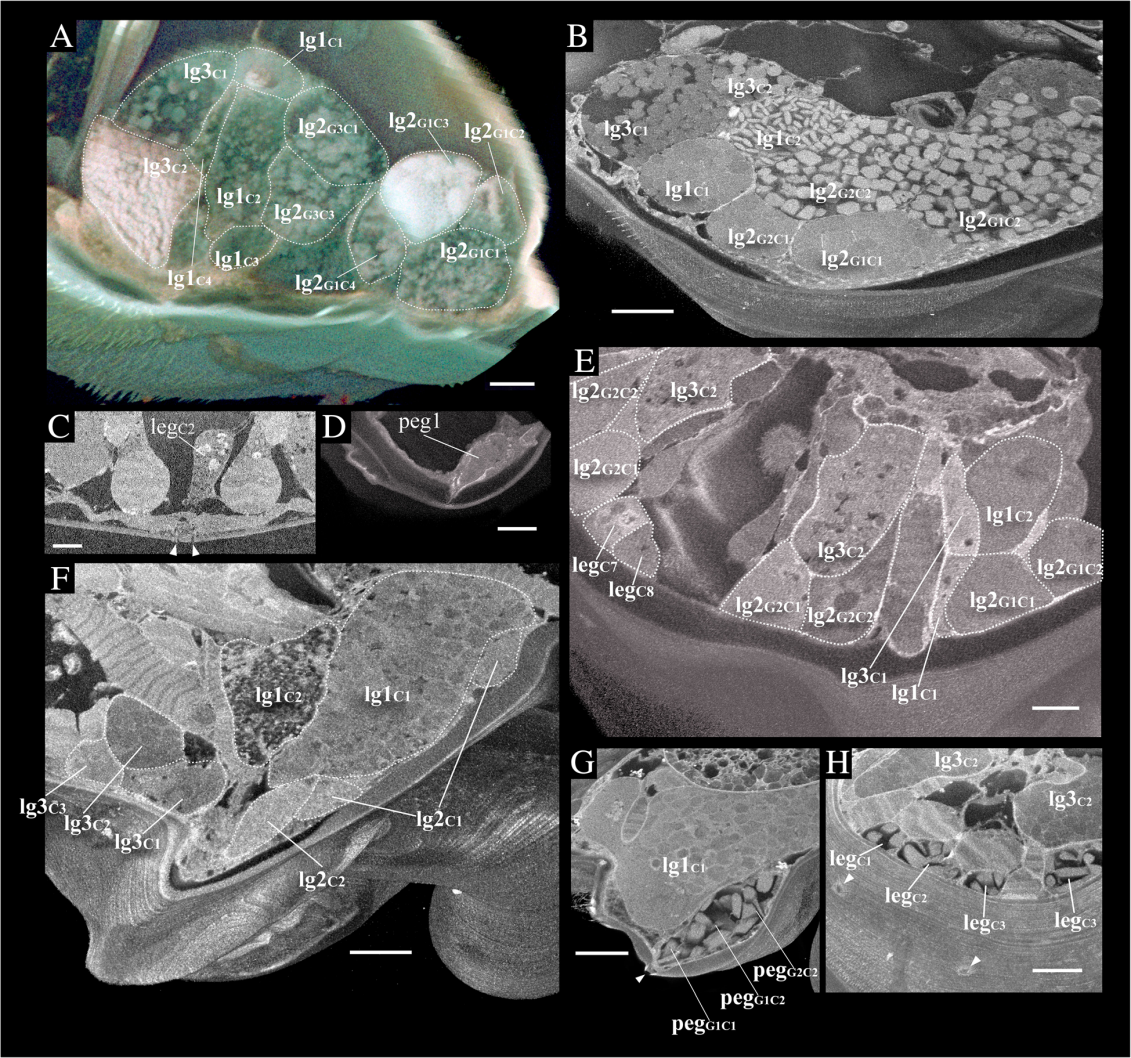
Ultrastructure of the gland cells based on volume rendering of two-photon excitation microscope (**a**) and SBF-SEM scans (**b-h**). **a** Coronal plane of labrum in *Disseta palumbii*. **b** Transverse plane of labrum in *Mesorhabdus gracilis*. **c** Magnified labral epidermal gland cell 2 in *M. gracilis*. **d** Magnified paradental epidermal gland cell 1 in *Heterostylites longicornis*. **e** Transverse plane of labrum in *H. longicornis*. **f-h** Transverse planes of labrum in *Heterorhabdus subspinifrons*. Arrowheads in C indicate openings of the epidermal gland cells. See abbreviations list Table 1 for gland names and abbreviations. Scale bars; 20 μ m for (**a**), (**d-h**); 30 μ m for (**b**); 10 μ m for (**c**)

Secretory granules in the gland cells appeared to vary among taxa and among the three gland types (Fig. 5). Granules in homologous types of gland cells (based on location) were not similar in shape and size (e.g., compare “lg3c1”and “lg3c2” in Fig. 5a; “lg1c1” and “lg1c2” in Fig. 5b; “lg1c2” and “lg1c1” in Fig. 5f). However, granule form of homologous gland cells also differed between individuals of the same species (Fig. 2), and even between sides of the same individual (Fig. 2b). Therefore, these observations, combined with inconsistent resolution due to technical limitations of contrasting and resolution, greatly limited the utility of granule form as a tool for making any inferences about gland function or homology.

A small, fourth type of gland — termed here Epidermal Gland — was found by the ventral side of the epidermis, with the duct opening on the ventral side of both the labrum and paragnath in *M. gracilis*, *H. longicornis*, and *H. subspinifrons* (Fig. 4b, c and d: represented in yellow). No such cells were seen in *D. palumbii*. Cell numbers were lowest in *M. gracilis* (2 cells; but paragnath epidermal gland might have been overlooked because of the limited scanning field), greater in *H. subspinifrons* (7 cells), and highest in *H. longicornis* (14 cells). In addition, arrangement of the labral epidermal gland cells was erratic and not always symmetrical (e.g., Fig. 4c and d).

Epidermal gland cells in *H. subspinifrons* contained distinctive spindle-shaped secretory granules (“peg” cells and “leg” cells in Fig. 5g, h). Unfortunately, the contents of these epidermal gland cells were unclear in other genera due to limited contrast and resolution (Fig. 5c, d and e).

### Muscle configuration and movement of mouthparts

Given the large differences in mandible form, the overall arrangement and attachment sites of muscles were surprisingly similar among the four genera examined (Fig. 4). These muscles are named based on their attachment sites or locations (Table 1). The only species-specific muscle we observed was in the highly derived carnivore *Heterorhabdus subspinifrons*, (“saggital labral muscle”, Fig. 4d, Table 1). This muscle was located at the posterior side of the labrum: one end attached just beside the opening of labral gland Type 1 and the other end attached near the esophagus opening (Fig. 4d: click on the view “Sagittal Labral Muscle insertions” in the interactive 3D-PDF).

**Fig. 6:**
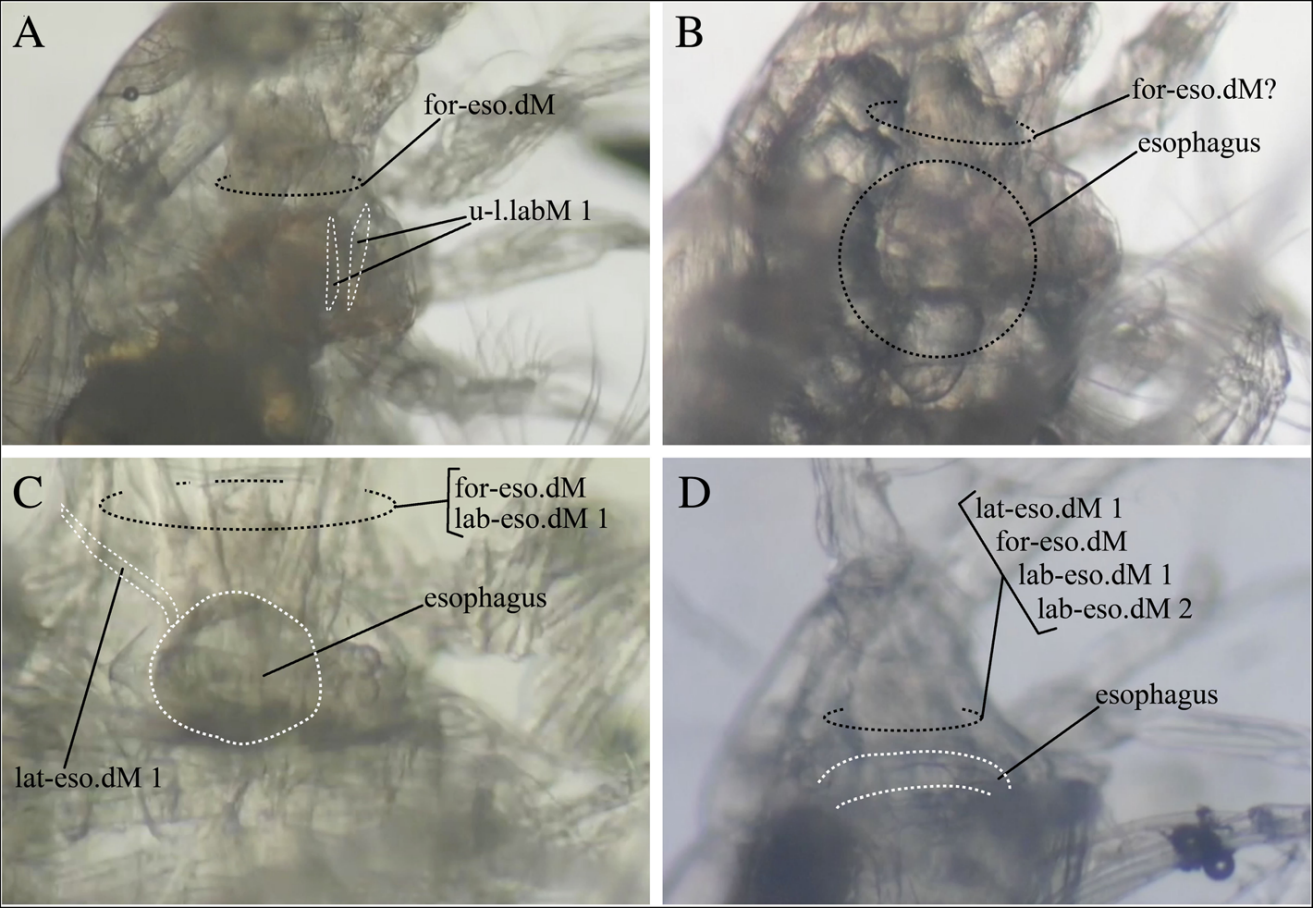
Frame-grab images from Additional file 2: Movie SM1 with structures of interest labeled. **a** *Disseta palumbii*. **b** *Mesorhabdus gracilis*. **c** *Heterostylites longicornis*. **d** *Heterorhabdus subspinifrons*. Black dotted circles identify the approximate area, and white dashed lines identify the exact boundaries, of the labeled characters. See abbreviations list and Table 1 for muscle names and abbreviations


In all four genera, masticatory movement of mandibles and cyclic muscular contraction within the labrum were synchronized soon after stimulation with a fine needle (Additional file 2: Movie SM1 A-D). In *Disseta palumbii* (particle feeder), cyclic contractions of the “Upper-Lower Labral Muscles 1” (u-l.labM1 in Fig. 3e) and the “Forehead-Esophageal Dilator Muscles” (for-eso.dM in Fig. 3e) were observed (Fig. 6a, Additional file 2: Movie SM1A). In *Mesorhabdus gracilis* (intermediate feeding mode), muscle bundles were not clearly recorded, but the “Forehead-Esophageal Dilator Muscles” (for-eso.dM in Fig. 3f) seemed to cyclically contract and lift up the esophagus area (Fig. 6b, Additional file 2: Movie SM1B). In *Heterostylites longicornis* (intermediate feeding mode), simultaneous cyclic contractions of the “Lateral-Esophageal Dilator Muscles 1” (lat-eso.dM1), the “Forehead-Esophageal Dilator Muscles” (for-eso.dM) and the “Labrum-Esophageal Dilator Muscles 1” (lab-eso.dM1 in Fig. 3g) created an expanding motion of the esophagus (Fig. 6c, Additional file 2: Movie SM1C). In *Heterorhabdus subspinifrons* (piercing carnivore), distinct muscles were not clearly recorded, but cyclic and coordinated contraction appeared to occur in the “Lateral-Esophageal Dilator Muscles 1” (lat-eso.dM1), the “Forehead-Esophageal Dilator Muscles” (for-eso.dM in Fig. 3h), the “Labrum-Esophageal Dilator Muscles 1” (lab-eso.dM1 in Fig. 3h) and the “Labrum-Esophageal Dilator Muscles 2” (lab-eso.dM2 in Fig. 3 h), which created an expanding motion of the esophagus (Fig. 6d, Additional file 2: Movie SM1D).

**Additional file 2: Movie SM1.** Mandible, muscle and esophagous motions in four heterorhabdid copepod species, all filmed at 30 frames per second. (A) *Disseta palumbii*, (B) *Mesorhabdus gracilis*, (C) *Heterostylites longicornis*, (D) *Heterorhabdus subspinifrons*. (MP4 50122 kb)


## Discussion

### Muscle homology among the four genera, and a novel muscle in carnivorous *Heterorhabdus subspinifrons*

Because of the highly conserved arrangement of muscles among the four heterorhabdid genera, putative homologues could be readily identified throughout (Table 1). Therefore, muscle arrangement itself did not appear to play a major role in the evolutionary shift among feeding modes. However, one unique muscle was found in the piercing carnivore *Heterorhabdus subspinifrons* (“Saggital Labral Muscles (s.labM)”, Fig. 4d). Labral gland Type 1 is assumed to be the main gland that charges venomous substances into the hypodermic needle-like mandibular fang [7]. This inference is supported by the position of the Type 1 gland opening, which is located exactly behind the charging pore of the mandibular fang (Fig. 4d). This structural arrangement of muscle and pores therefore suggests that muscle contraction enhances the efficiency of venom charging by adjusting the position of the pores relative to the base of the fang.

### Revised homology hypotheses for the glands

Ohtsuka et al. [9] hypothesized that the Type 1 gland in *Heterorhabdus subspinifrons* is a specialized form of one of the two Type 3 glands that exist in other heterorhabdid species. To be consistent with our homology hypothesis, we revised this terminology, and adopt the term Type 1 for all species examined here (see Figs. 1 and 3). Our hypothesis of gland homology, consistent with traditional criteria for structural homology [28], is based on the unambiguous spatial relationships of gland openings: Type 2 are located at the lateral tip of the labrum and contain 2–3 openings; Type 1 are located beside the Type 2 opening complex; and Type 3 are located at the most medial part of the labrum compared to the other openings. Although we did observe differences among species in the contents of putatively homologous glands (Fig. 5), gland contents also differed significantly between individuals of the same species and sides of the same individual (Fig. 2). Therefore, apparent differences in gland contents among species in Fig. 5 are unlikely to be informative phyologenetically and do not impair our homology inferences.

As noted in the results, we did find a few differences in cell numbers and structures between our results and previous observations [7]. These differences might be due to the different species we used, but they are more likely due to the greatly enhanced spatial resolution of our 3-dimensional analysis. Nonetheless, these differences do not affect our homology inferences.

Finally, the previously reported “paragnathal gland” (presumed to be associated with a “*pore in the ventro-medial region of the posterior surface of each paragnath*”; visible in Fig. 3a of Nishida and Ohtsuka [7]) we now consider to be a synonym of the paragnathal epidermal gland defined here.

### Venom-assisted feeding in Viper copepods

Venom-assisted feeding is a complex adaptation that requires concordant evolution in two novel functional domains: novel physiology (pharmacologically active venom compounds [18]) and novel morphology (venom delivery system [11]). Despite this complexity, venom-assisted feeding has evolved multiple times in arthropods, including chelicerates, myriapods and insects [12]. Surprisingly, despite their immense diversity and success in marine and aquatic systems, venom-assisted feeding appears to be exceedingly rare in Crustacea. The only definitive case occurs in remipedes [12]. Envenomation is suspected in two parasitic crustacean groups (branchiuran fish lice and siphonostomatoid copepods) as well as two free-living taxa (caprellid amphipods and heterorhabdid copepods) [12], but pharmacological confirmation is still required.

In the piercing carnivorous copepod, *Heterorhabdus subspinifrons,* cells of the Type 3 labral gland are tightly enveloped by multiple muscles (for-eso.dM, lab-eso.dM 1–4 and u-l.labM2; Figs. 3h, 4d). This arrangement implies that muscular contraction squeezes the gland to eject secretions. Ejection of secretions via muscular contraction appears to be a common strategy in many animals: venom release in cone snails [19]; venom gland discharge in elapid and viperid snakes [20]; silk ejection from the antennal exopod in ostracods [21]; and venom ejection from the head of specialized soldier termites [22]. Furthermore, these muscles in *H. subspinifrons* all contract cyclically during experimentally induced mastication (Fig. 6d, Additional file 2: Movie SM1D), so presumed venom ejection likely occurs simultaneously with mastication.

Three of our observations suggest that the piercing carnivore *Heterorhabdus subspinifrons* injects a poison or venom into its prey via the hollow fang on its mandible. First, one gland type (Type 1) is greatly enlarged. Second, the opening to that enlarged gland shifted to lie at the base of the hollow fang (Fig. 3d). Third, secretions from the Type 3 gland in *H. subspinifrons* may have a specific role in carnivorous feeding, because the enveloping arrangement of muscles around the glands does not occur in the other heterorhabdid species examined (Fig. 4). However, pharmacological evidence would be required to show definitively that the secretions are a venom and not mucus or some other salivary secretion.

### Minor morphological change supported a major radiation of feeding strategies

Despite drastic functional changes — from particle feeding with a mundane mandible to carnivorous feeding with a sophisticated piercing-injection system — the overall morphological units and their arrangement are strikingly similar among the Viper copepod species examined here. This similarity implies that differential use of the mandible, for simple mastication or for venom injection, can be accomplished by a slight modification of cuticle structure and minor modification of muscle structure. Other examples of great functional innovation in pancrustaceans follow a similar principle, where minor morphological modifications facilitate significant functional change. First, in some highly derived snapping-shrimp genera, like *Alpheus* and *Synalpheus*, minor changes in muscle structure (e.g., subdivided claw-closer muscle) maximize the efficiency of the latch-releasing motion before snapping [23]. Second, a similar evolutionary sequence of muscle subdivision to control latch release is seen in *Anochetus* trap-jaw ants [24]. Third, muscles in the suction disc of adult parasitic branchiuran Crustacea are identical to those in the larva that control ordinary appendage-like motion (the larval mouthpart appendage is the anlagen of the suction disc), except for two newly acquired muscles — “circular sucker muscle” and “disc rim muscle” — that both minutely adjust the shape of the sucker to attach it to the host surface in the most efficient way [25]. This evolutionary tendency — for seemingly minor but functionally significant adjustments of form to a novel function — is comparable to acquisition of the novel “sagittal labral muscle” in *Heterorhabdus subspinifrons*. Because that muscle, which adjusts the gland opening to the pore of the mandibular fang, is the only newly acquired muscle associated with piercing carnivory, it may enhance efficiency of charging the fang with liquid. Since this efficiency may have critical role in the newly acquired “fang” function of the mandible, this small muscle may play an important role in the new carnivorous feeding strategy. The evolutionary shift of the Type 1 gland opening to lie at the proximal end of the mandibular fang in *H. subspinifrons* (unlike other taxa), also likely enhances the efficiency of injection.

Such drastic changes of this function-adaptation complex, enhanced by minor morphological change, may facilitate invasion of wholly new adaptive zones and potentially explosive diversification in harmony with body mituarization [26]. The remarkably high diversity of heterorhabdid copepods that utilize piercing carnivory ([10], Fig. 1) implies that functional transformation of feeding structures may have greatly accelerated the rate of evolutionary diversification.

### Three-dimensional visualization of small animals and the “renaissance of morphology”

This study also illustrates the great power of new imaging tools, and sophisticated 3D visualization techniques, to help understand complex morphologies, particularly in the small creatures that make up the vast majority of animal diversity. These advances have led to a “renaissance of morphology” [16, 17].

The difficulties of 3D imaging in small animals were overcome by using two advanced imaging techniques: serial block-face scanning electron microscopy (SBF-SEM) and two-photon excitation microscopy. SBF-SEM uses a robotic ultramicrotome-embedded within a scanning electron microscope. It is a major advance over confocal laser scanning microscopy (CLSM: appropriate specimen thickness roughly 10–150 μm) and micro-computed tomography (micro-CT: appropriate specimen size roughly 1 mm-20 cm) because it permits 3D reconstruction of meso-scale structures (roughly 100–1000 μm) at nanometer resolution [14]. Two-photon excitation microscopy also yields nanometer resolution of meso-scale structures up to one millimeter depth-of-field [15].

The 3D information contained in the high-resolution image stacks were made comprehensible and presentable by advanced 3D visualization techniques. First, each discrete morphological element (specific muscle or gland) can be segmented out of each plane of an image stack (e.g., see outlined regions in Fig. 5) so that it can be rendered in three dimensions and assigned an informative color and shading (e.g., Fig. 3e-h). But such 3D renderings can still be difficult to interpret from 2D perspective images where many component elements are involved (e.g., Fig. 3e-h). The limitations imposed by 2D representations of 3D renderings are overcome entirely by interactive 3D models that can be incorporated directly in pdf files (e.g., Fig. 4). These 3D interactive models give the viewer extraordinary viewing power: 1) virtually unlimited zoom and pan capability, 2) the ability to look at one subset of structures at a time (e.g., only muscles or only glands), and specific items in each subset, via logically structured hierarchical groupings of elements (see Additional file 1: Figure S1 for viewing tips), 3) the ability to examine specific pairs or specific sets of structures in isolation (e.g., the relations of specific muscles (for-eso.dM, lab-eso.dM1–4 and u-l.labM2)) associated with the Type 3 labral gland in *Heterorhabdus subspinifrons*) by excluding all other structures. Such selective viewing was vital to understanding the 3D spatial relations of component parts in the piercing carnivore *H. subspinifrons*. It also allows readers to judge for themselves these relations free from any author prejudice.

## Materials and methods

### Collection and imaging

Specimens were collected off the Nansei Islands, southwestern Japan in 2016–2017, by oblique towing of a large-diameter plankton net (ORI, diameter 1.6 m; mesh size 0.33 mm) between 0 and 728 m depth with the vessel TRV *Toyoshio-maru*, Hiroshima University. Detailed localities are: *Disseta palumbii*- east of Nakanoshima Island (29°31.412′N, 130°37.296′E); *Heterostylites longicornis*- east of Tanegashima Island (30°13.218′N, 131°09.252′E); *Heterorhabdus subspinifrons*- east of Tanegashima Island (30°52.168′N, 131°34.897′E); *Mesorhabdus gracilis*- east of Okinoerabujima Island (27°10.857′N, 129°03.307′E).

In preparation for observations by SBF-SEM, individuals were fixed with 2% glutaraldehyde and 2% paraformaldehyde in 0.15 M cacodylate sodium buffer with 2 mM CaCl_2_ (pH 7.4) for 5 h at 4 °C, then decalcified in 10% EDTA in water for 2 days at 4 °C. The specimens were post-fixed with 2% osmium tetroxide and 1.5% potassium ferrocyanide in the same buffer for 2 h at room temperature. They were incubated in 1% thiocarbohydrazide for 30 min at room temperature, and fixed again with 2% osmium tetroxide in water for 1 h at room temperature. En bloc staining was performed with 1% uranyl acetate for 3 h at room temperature and then with Walton’s lead-aspartate solution (20 mM, pH 5.5) for 60 min at 60 °C. The specimens were washed with cacodylate buffer or distilled water between each step described above. Each specimen was a) dehydrated by a graded ethanol series (30–100%) at 4 °C with 30 min for each step, b) transferred to 100% acetone for 1 h, and c) incubated in a graded Durcupan resin series (25, 50, 75, 100% using acetone as a solvent) in a vacuum chamber for 12 h at each step. The resin was allowed to polymerize at 60 °C for 3 days. Trimmed resin blocks were glued onto an aluminum SBF-SEM rivet with conductive epoxy resin (SPI Conductive Silver Epoxy; SPI Supplies and Structure Prove, Inc., West Chester, PA, USA), and coated with gold using an ion coater. Scanning electron microscopes (SIGMA/VP and MERLIN, Carl Zeiss Microscopy, Jena, Germany), equipped with an in-chamber ultramicrotome system and a back-scattered electron detector (3View; Gatan Inc., Pleasanton, CA, USA), were used to slice and image each specimen as described previously [27]. The serial-section image stack was acquired in an automated fashion by using Gatan Digital Micrograph software.

In preparation for observations by the multiphoton microscope (Leica TCS SP8 MP), specimens were fixed in Bouin’s solution, dehydrated in an isopropanol series, and then mounted on slides using a 2:1 mixture of benzyl benzoate and benzyl alcohol for clearing. Specimens were imaged using autofluorescence, so excitation wavelength, detected emission wavelength range, etc., were adjusted individually for each specimen to obtain maximum brightness and contrast.

### 3D visualization and videography

Image stacks from SBF-SEM were automatically aligned using the registration plug-in “Register Virtual Stack Slices” in Fiji/ImageJ software package (http://fiji.sc/Fiji). Surface and volume renderings of the scanned data were performed using IMARIS 7.0.0 (Bitplane AG). Objects for the 3D-pdfs were exported as vrml format. File sizes were reduced by MeshLab (http://www.meshlab.net/), and then exported as u3d format. Files were arranged using Deep Exploration (Right Hemisphere) and re-arranged by Adobe Acrobat Pro (Adobe) to create 3D-pdf files.

To video mouthpart motion, living copepods were briefly semi-dried and attached to a glass dish with cyanoacrylate glue on the dorsal side of the metasoma and the dish was then filled with seawater. Positioned copepods were stimulated to move their mouthparts using a needle. A video camera EX-F1 (CASIO, Japan) was used to record mouthpart behavior.

## Additional files

Additional file 1: **Figure S1.** Instructions for how to use the viewing functions of the interactive 3D-pdf in Fig. 4. 1) Click any panel you want to view. 2) Click model tree icon (A) to reveal operation windows (B) and (C). 3) Window B shows the heirarchical tree diagram of morphological characters defined in this paper. Click the arrowhead to the left of each to reveal subcategories, and click each checkbox to hide/unhide each specific character. 4) Click “view”s (C) to view the specified perspective of the characters selected in (B) as described and instructed in the main text. (JPG 877 kb)

## Muscles

esoS: Esophageal Sphincters
for-eso.dM: Forehead-Esophageal Dilator Muscles
lab-eso.dM: Labrum-Esophageal Dilator Muscles
lat-eso.dM: Lateral-Esophageal Dilator Muscles
parM: Paragnath Muscles
s.labM: Saggital Labral Muscles
t.labM: Transverse Labral Muscle
u-l.labM: Upper-Lower Labral Muscles

## Glands (X identifies gland type number, Y identifies a cell group number, and Z identifies a numbered cell within a gland or group)

leg: Labral Epidermal Glands
leg_CZ_: Labral Epidermal Gland Cell Z
lg: Labral Glands
lgX: Labral Gland Type X
lgX_CZ_: Labral Gland Type X Cell Z
lgX_GYCZ_: Labral Gland Type X Group Y Cell Z
peg: Paragnathal Epidermal Glands
peg_CZ_: Paragnathal Epidermal Glands Cell Z
peg_GYCZ_: Paragnathal Epidermal Glands Group Y Cell Z
