# Supplementary figures and images for: Evolutionary transformation of mouthparts from particle-feeding to piercing carnivory in Viper copepods: Review and 3D analyses of a key innovation using advanced imaging techniques
Source: Front Zool. 2019 Aug 22;16:35. doi: 10.1186/s12983-019-0308-y (PMC6704645; doi:10.1186/s12983-019-0308-y)

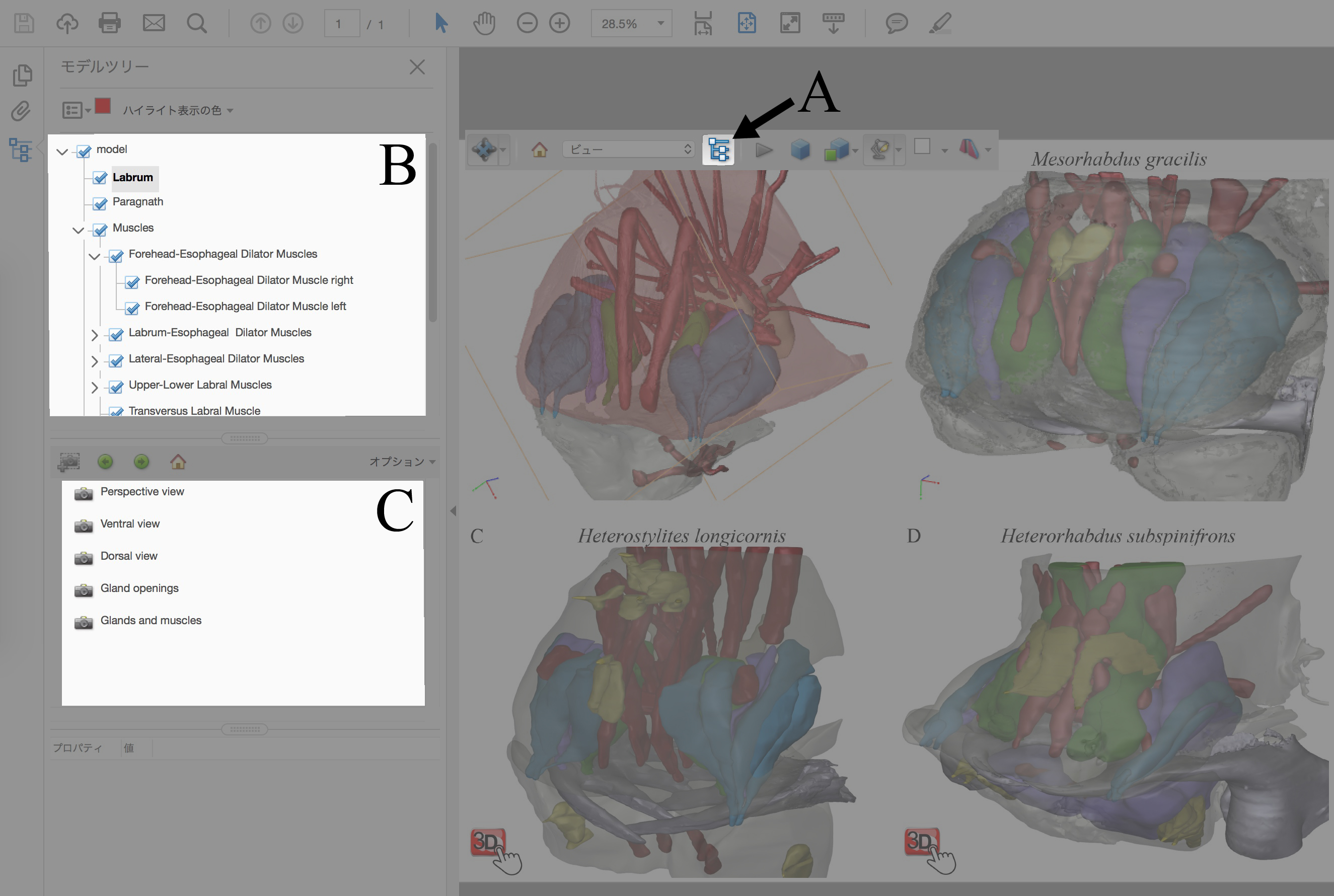

Supplement: Supplementary file 2 — Figure S1. Instructions for how to use the viewing functions of the interactive 3D-pdf in Fig. 4. 1) Click any panel you want to view. 2) Click model tree icon (A) to reveal operation windows (B) and (C). 3) Window B shows the heirarchical tree diagram of morphological characters defined in this paper. Click the arrowhead to the left of each to reveal subcategories, and click each checkbox to hide/unhide each specific character. 4) Click “view”s (C) to view the specified perspective of the characters selected in (B) as described and instructed in the main text. (JPG 877 kb) [file 12983_2019_308_MOESM1_ESM.jpg]
